# Researching into Chinese university students’ mental health in the post-pandemic era – problems and causes

**DOI:** 10.3389/fpsyg.2024.1393603

**Published:** 2024-06-25

**Authors:** Xuan Ning, Xiaoyu Luo, Sijia Guo

**Affiliations:** ^1^Beijing Normal University-Hong Kong Baptist University United International College, Zhuhai, China; ^2^Hong Kong Polytechnic University, Kowloon, Hong Kong SAR, China; ^3^Dalian Maritime University, Dalian, China

**Keywords:** Chinese university students, mental health, post-pandemic era, focus groups, qualitative research

## Abstract

**Introduction:**

Mental health challenges have still been widely pervasive among Chinese university students after the outbreak of the COVID-19 pandemic. This article aims to explore mental health challenges encountered by Chinese university students in the post-pandemic era and explain causes to these challenges using a qualitative approach.

**Methods:**

We conducted six focus group interviews with university students in Zhuhai, China, and altogether 61 students including 43 female students, and 18 male students participated in the study.

**Results:**

Our results indicate that sleep difficulties, anxiety, and stress are the three primary mental health challenges experienced by students. Academic pressure, social influence including peer pressure and pursuit of social acceptance, and pandemic related policies and measures are the causes to the above mental health challenges.

**Discussion:**

The results of this study will inform the development of mental health promotion, intervention, and education activities for university students to bolster their resilience and cope with mental health problems in the post-pandemic era. Meanwhile, our results could illuminate the services stakeholders provide to university students in the future.

## 1 Introduction

The global COVID-19 pandemic has brought far-reaching implications for people’s mental health, and it has still been severely affecting individuals’ mental health even after the outbreak stage of this pandemic (Rudolph and Zacher, 2020). Among the groups of people that have been greatly affected by this pandemic, university students in China pose themselves to be a group that is worth specific attention considering their increased mental health challenges and decreased level of mental health during the pandemic (Chen et al., 2020; Zhao et al., 2023).

Research has illuminated an upward trajectory of depressive symptoms among Chinese college students (Tang et al., 2018; Dong and Li, 2020). The prolonged psychological distress experienced by these students has been associated with diminished academic attainment, anxiety, depression, and suicidal behavior (Lu et al., 2015). Over the past decade, the incidence of mental health issues such as depression and suicide has been alarmingly high within the Chinese university student demographic. According to some studies, the prevalence of mental health disorders among Chinese university students ranges from 20 to 30% (Luo et al., 2021).

For Chinese university students, the alterations and challenges instigated by the pandemic have directly engendered repercussions on their psychological well-being. A large-scale survey reported that about 7.7% of college students had depressive symptoms during the pandemic (Chen et al., 2020). To date, previous research has established several common symptoms among university students in China, such as depression, anxiety, acute stress, and insomnia (Li et al., 2021). During the post-pandemic era, insomnia and depression are common symptoms of university students in Wuhan, the first city to suffer from the early stages of COVID-19 (Duan et al., 2022).

Preceding studies have substantiated that the negative effect precipitated by the COVID-19 outbreak and the attenuation of individual resilience consequent to persistent stressors can exacerbate the vulnerability of an individual’s the physical and psychological well-being (Ren et al., 2020). In the post-epidemic era, despite the gradual restoration of the physical health landscape, the deleterious ramifications on mental health stemming from the pandemic may endure or potentially intensify (Wang et al., 2022). Subsets of university students persist in grappling with depression, anxiety, acute stress, and insomnia subsequent to their return to school. Additionally, the degree to which COVID-19 exerts its influence on an individual’s day-to-day operational capacity evinces a positive correlation with compromised mental well-being (Wang et al., 2021). Students were concerned about the infection, which was related to the shortage of medical supplies, stressful quarantine, and public stigmatization in the early stages (Ding et al., 2023). Another factor that affected students was COVID-19-related regulations implemented by governments and universities. To restrict the spread of the virus and ensure public health, governments took the segregation and quarantine policy, and universities in China were sometimes under strict lockdown policies, which led to short- and long-term impacts on the education and mental health of university students (Elmer et al., 2020). The unpredictability of students’ life plans had also risen sharply, together with the update of the public health policies reflecting the fluctuation of the pandemic situations, which caused students’ stress and anxiety (Aristovnik et al., 2020; Huang et al., 2023). Existing longitudinal studies find that the level of stress, anxiety, and depression of Chinese university students has increased during the post-lockdown period (Li et al., 2020). Under these policies, students were faced with higher risks of social isolation and consequent mental disorders as a result of being quarantined and, thus, reducing their social activities, which became a vicious cycle (Aristovnik et al., 2020; Chen et al., 2020; Xu et al., 2022). As Chinese university students primarily lived on campus, it was hard for them to strictly follow the related university COVID-19 regulations while some of their peers did not, which would incur some students extreme behaviors (Ding et al., 2023). The dormitory relationships were more sensitive during this period as roommates had to stay together for a longer time in a state of stress with a lower sense of social support, which caused heightened stress of university students (Ma et al., 2020).

Another significant change for Chinese university students was switching their study mode from offline to online, which was a sudden shift in their learning patterns (Sahu, 2020). This abrupt shift created extra discomfort and challenges for students, which resulted in an increase in their psychological stress due to isolation and concentration problems (Yeung and Yau, 2022). Self-efficacy became one of the determining factors for online studying for students who could not go back to campus because of COVID-19 (Hong et al., 2023). Students needed time to adapt to the new study mode and adjust their learning habits accordingly, which would bring challenges to students’ mental health (Curelaru et al., 2022; Li and Che, 2022). Some students had concentration problems while learning online (Aristovnik et al., 2020). Students felt a decrease in peer interaction and an increase in isolation, and the isolation problem was further deteriorated by losing normal in-person contact with professors and peers (Ding et al., 2023).

Given the complicated factors that could induce Chinese university students’ mental health challenges during the post pandemic era, a holistic understanding and vigilance concerning the psychological well-being of Chinese university students is indispensable. While the preliminary stages of the pandemic have attracted increased research focus, relatively limited inquiry has been directed towards the post-COVID-19 period. Additionally, extant research within this realm predominantly assumes a quantitative orientation, necessitating greater incorporation of qualitative methodologies. To fill these research gaps, the present study aims to delineate the mental health problems encountered by Chinese university students during the post-pandemic era, accompanied by an exploration of underlying causative factors. This research holds pragmatic significance in unraveling the current psychological status of college students, proffering guidance for their improved adaptation to post-pandemic academic and personal realms. Furthermore, this study offers guidance for both Chinese and foreign universities to formulate interventions addressing the contemporary psychological state of university students, thereby facilitating their ability to navigate the manifold changes and challenges heralded by the post-pandemic era.

## 2 Methods

Between April and October 2022, data collection was conducted. During this timeframe, unwavering implementation of the general policy of “dynamic zero” was in effect, and the pandemic prevention and control policy adhered to the principles of taking prevention as the main focus, integrating prevention and treatment, adhering to scientific methods, and implementing hierarchical classification. The university at which data collection took place implemented a range of measures, encompassing closed-campus management, intensified scrutiny of various gatherings, and avoidance of large-scale gatherings unless deemed essential. Moreover, the university established nucleic acid testing facilities, mandating a minimum 72-h testing period for individuals entering or exiting campus premises.

To maximize the participation of students, a combination of online and offline strategies was employed. For online recruitment, pertinent information concerning the research project was disseminated within WeChat groups established to propagate health promotion information among university students. The selection of specific WeChat groups was conducted in consultation with student leaders. Concurrently, offline efforts included displaying posters containing study information at canteen entrances and student dormitories to facilitate recruitment. Additionally, snowball sampling was utilized to engage students through participants who had already taken part in the initial round of focus groups. All students expressing interest in participating were instructed to contact the project coordinators directly. The recruitment materials explicitly stated that participation was entirely voluntary and dependent on the student’s willingness. Participants were assured of confidentiality, anonymous coding of their identities, and the option to withdraw from the study at any time. Group rules were stated at the beginning of each focus group discussion to set up a safe and open atmosphere for participants. The information on mental health hotlines and on-campus counseling support was provided to all the participants in case they might develop any negative feelings during or after the focus group discussion. Ultimately, 61 students were recruited and participated in the 6 focus groups, comprising 43 female students and 18 male students (please see Table 1 for detailed focus group information). Participants’ ages ranged from 18 to 22 years, including 15 first-year students, 16 s-year students, 14 third-year students, and 16 fourth-year students who studied in the major programs including social work, sociology, applied psychology, food science, accounting, finance, and computer science.

**Table 1:** Focus group information.

	**Number of participants**	**Mode**	**Number of facilitators**	**Length**	**Date**
Focus group 1	Total: 12 (8 males, 4 females)	Offline	3	1 h 28 min	April 29, 2022
Focus group 2	Total: 9 (5 males, 4 females)	Offline	3	1 h 30 min	May 19, 2022
Focus group 3	Total: 13 (1 male, 12 females)	Offline	3	1 h 35 min	September 21, 2022
Focus group 4	Total: 13 (1 male, 12 females)	Offline	3	1 h 25 min	September 23, 2022
Focus group 5	Total: 7 (2 males, 5 females)	Offline	3	1 h 39 min	September 30, 2022
Focus group 6	Total: 7 (1 male, 6 females)	Offline	3	1 h 46 min	October 14, 2022

Informed consent was acquired from each participant prior to their engagement in the focus groups. The interviews were conducted in Mandarin, which served as the participants’ primary language. On average, each focus group session extended to approximately 2 h. Facilitation of the focus groups involved two to three individuals, who comprised research team members or graduate trainees. The research members and trainees were from mental health-related disciplines including public health, social work, sociology, and psychology. To guide the focus group discussions, an interview guide was employed by the research team based on the literature review and current research gaps, aiming to delve into participants’ perceptions of mental health and mental illness, their insights into university students’ mental health issues and requirements, their perspectives on the underlying causes of university mental health problems, and their recommendations for this research endeavor. All of the focus group discussions took place in a lounge designed for students’ activities located in XX university (omitted the exact name here for the anonymous manuscript), which was convenient for participants to go to but meanwhile retained restrained access so that non-invited students would not be able to enter this lounge during the focus group discussions. All of the discussions were audio-recorded and subsequently transcribed verbatim in the Chinese language for data analysis purposes. The transcribed interviews were cross-validated by the research team members to ensure accuracy. Subsequently, the Chinese transcripts were translated into English by the first author and reviewed by the second author, both of whom possess bilingual proficiency and have been deeply involved in the research processes of this project.

The data analysis process was guided by McCracken’s (1988) thematic coding approach utilizing NVivo software. First, the first two authors who were engaged in the data collection process and who conducted undergraduate studies in China familirazed themselves with the data by repetitively reading the transcripts. Then, they meticulously examined the transcipts independently to establish a comprehensive understanding of the participants’ narratives. Subsequently, a line-by-line open coding technique was applied to identify key ideas expressed by the participants and concepts relevant to the research inquiries. An initial list of codes were identified from this inductive approach. These open codes were then organized into coherent themes based on their similarities and distinctiveness. A list of potential themes was identified, then the two coders evaluated the fitness of the extracted codes to a particular them that they made up, and then whether all the themes generated could encapsulate all of the collected data. A collaborative meeting between the two coders was conducted to deliberate, compare, and interpret the finalized themes. For the disagreements and ambiguity existed between the results of the two coders, in-depth investigations of the data instances coded were carried out to engage the coders to reflect on the assumptions that caused disagreements between them, as these assumptions might potentially shape their perspectives and limit their data interpretation. This two-step data analysis procedure facilitated the integration of diverse analytical perspectives from team members, ultimately yielding the most robust explication of the participants’ narratives. Consequently, this analytical process enhanced the depth of insights into the pertinent issues and bolstered the credibility of the research findings (Shenton, 2004). The thematic analysis enabled a comprehensive representation of the participants’ accounts, thereby augmenting the contextual transferability of this study (Houghton et al., 2013). The study has been granted ethical approval by XX (omitted the exact name here for the anonymous manuscript). The targeted recruitment objective encompassed 6 focus groups, each consisting of approximately 8 individuals. Inclusion criteria necessitated that participants should be above the age of 18 and enrolled at XX university (omitted the exact name here for the anonymous manuscript) located in Zhuhai, China.

## 3 Results

### 3.1 Mental health problems

#### 3.1.1 Sleep difficulties

Students reported having sleep difficulties related to certain time points: the beginning of a new semester, the end of a semester, and the beginning of vacations. Many participants experienced sleep difficulties at the beginning of a new semester, as indicated by them, this was due to the adaptation issues of adjusting their vacation life routine to college life routine, and they were also away from their family and developed a sense of nostalgia.

I left my hometown and parents to start college in a faraway city, and I felt so uncomfortable and nervous to be in a totally strange place, knowing no one and nothing. I had very serious problems going to sleep during that period and had to rely on sleeping pills to fall asleep each night. (Focus group 2 #, Student 9#, female).

Except for the beginning of a semester, the end of a semester with multiple final examinations coming in at the same time is also a critical time point when lots of university students develop sleep difficulties.

I always have problems going to sleep, but when it is during the final season, my sleep difficulties will further deteriorate. If there is a final examination tomorrow, then I will not be able to fall asleep tonight. What is worse, sleeping pills are not helpful at all under this circumstance. (Focus group 1 #, Student 1#, male).

Students have winter and summer vacations every year during their college studies, and some participants indicated that they had sleep difficulties during their vacations due to the loose schedule of their lives. The vacancy in students’ lives made them feel guilty that they had wasted their time and therefore could not catch up with their peers who might use the vacation time to do internships. Students who had a loose schedule stated that they wanted to pack up their schedule with study-related activities, however, they lacked the momentum to carry out those planned activities by not having a collective learning environment as they did in university.

I frequently have sleep difficulties, and different from my peers who have this problem during the academic semester, I encounter this issue when I go back home for vacations. I could lie on my bed for a couple of hours without feeling sleepy even a little bit. Rather than having too much on my mind that I cannot fall asleep, I have nothing on my mind and it makes me feel quite hollow and guilty for wasting my time. (Focus group 1#, Student 4#, female).

#### 3.1.2 Anxiety

Participants indicated that anxiety was another mental health challenge faced by them. Participants perceived that their anxiety came from the life changes caused by measures to contain the COVID-19 pandemic. The Chinese government has implemented various measures to control the spread of COVID-19. These measures included strict lockdowns, mass testing, contact tracing, and mandatory quarantine. Moreover, a system of health codes that allowed individuals to show their health status and travel history was implemented, which was used to restrict movement in areas with high infection rates. These were effective measures to slow the spread of COVID-19 and reduce the infection rate. Nonetheless, they brought drastic changes and uncertainties to students’ lives in terms of study mode, social mode, and plans after graduation.

Online learning was the major learning mode during the outbreak of COVID-19, and it was still the case when a couple of new cases were confirmed in an area before China lifted its restrictions on COVID-19. The majority of the participants found they had relatively low learning efficiency and inconvenient when it came to work that required group collaboration and felt quite anxious.

I had to take online courses for 3 days after I came back to university, as there were newly confirmed COVID-19 cases in my hometown within the past 7 days, so I was asked to conduct a 3-day mandatory quarantine in a designated hotel by the local government. It was very troublesome to take online courses at the beginning of a semester when I needed to find groupmates to do group projects. While being trapped in a hotel room by myself, I was also underprivileged to find good students to form a group as I was not able to know who was in that class if I did not go to offline classes. (Focus group 3#, Student 11#, female).

Participants also complained that the sudden switch from offline learning to online learning rendered a drop in their academic performance, which made them extremely concerned and worried.

I hate online classes, and I had never taken online classes before the pandemic. However, after the pandemic outbreak, everything was switched online, and my grades dropped drastically because I had a hard time adjusting to learning online. (Focus group 3#, Student 1#, female).

In addition to the learning mode, the way participants socialize with other people was changed due to COVID-19. Instead of hanging out with their friends in person, participants spent more time on their online social networks to maintain contact with their friends. Socializing with people online was different from in-person communication, especially for students who had social anxiety disorders. As students could create different digital identities in the virtual world, and communication did not require eye contact or instant response like in-person communication did. Hence, they could be more flexible and less rigid when it comes to online socialization, which might hinder their ability to socialize with other people in person. Therefore, when students resumed offline learning and in-person socialization, some participants encountered more challenges in engaging in social life.

When I went back to school, I felt extremely anxious whenever I talked to my peers, especially to the people that I barely knew. It seemed that I was very talkative to other people, but only I knew it was because I was so nervous that only keeping talking would cover my anxiety when socializing with other people. (Focus group 3#, Student 1#, female).

#### 3.1.3 Stress

Another mental health challenge reported by participants was stress, which resulted in poor physical health, lack of stamina, and sleep difficulties.

When I am under severe stress, I lack stamina, which is the condition I have been in recently. I cannot sleep very well at night, and my study efficiency is low, either. Besides, the stress reflects on my poor physical health, too, as I have not had any appetite for eating lately, and I even do not feel hungry even though I do not have one meal at the end of the day. (Focus group 6#, Student 7#, male).

One participant mentioned that his friend did self-harm to himself to reduce the overwhelming stress.

My friend scratches his wrist with a knife every time he feels stressed, which leads to multiple scars on his wrist. I tried to stop him from hurting himself like this, but he said that this is a good outlet for him to vent his stress. (Focus group 5 #, Student 2 #, female).

The implementation of school closures and related measures in response to the pandemic significantly disconcerted students, contributing to their experienced stress. The confinement imposed by school closures restricted students’ space, and opportunities for engaging in physical activities, and other pursuits, resulting in students’ increased time spent on social media platforms, and posing challenges in finding suitable avenues for stress reduction. As a consequence, students might encounter additional difficulties in self-adjustment and emotional management, which culminated in the accumulation of students’ persistent stress.

The closure of my university has greatly changed our behaviors. Previously, when we could leave the campus, my friends and I usually took a walk or hit different interesting places to let off some pressure. But now, confined to school premises, the options to de-stress are limited. (Focus group 1#, Student 3#, male).

During that extended university closure, I felt like I was losing my mind because I had no idea how to fill my time. It was driving me crazy. (Focus group 4#, Student 9#, male).

One participant reflected that exposure to negative news on social media had the potential to induce stress among university students.

I had more time spent on social media as the school closed, and there was not much to do. The news regarding the pandemic was everywhere, and the negative coverage of the pandemic really made me addicted to reading it. The more I was concerned about the pandemic, the more I read about the coverage. And I got more and more stressed out when I realized that I could not change the situation. (Focus group 1#, Student 3#, male).

This is an information explosion age with all sorts of news that are hard for my peers and me to distinguish, and we are immersed with news regarding the pandemic all day long, which creates a dull and severe atmosphere and makes us feel pressed all the time (Focus group 6#, Student 3#, female).

Moreover, students who had prior mental health challenges had more tough situations than their peers who did not experience any mental health challenges before the pandemic.

### 3.2 Causes of students’ mental health challenges

#### 3.2.1 Academic performance

Academic performance poses severe challenges to participants’ mental health, which leads to their mental health disorders of anxiety and stress. Challenges students encountered in their study and peer pressure were two aspects of academic performance that accounted for the reasons why participants felt anxious and stressed.

First, challenges students encountered in their study, such as being unable to absorb new content, having trouble following their instructors during class, experiencing difficulties completing assignments, and so forth, imposed stress on their mental and physical health.

Studying in university is different from high school. The courses I take in university would not require me to submit objective answers like I used to when I was in high school. Therefore, I have had a difficult time adapting to university study, which has resulted in my unsatisfactory academic performance. I have been so anxiously concerned about my grades that I have lost my appetite and sleep quality for a long period. (Focus group 1#, Student 5#, female).

Participants stated that their stress levels and anxiety increased when mid-term and final examinations approached, as they were especially pressed by the challenges in their study.

I always feel extremely stressed out during examination season. One or two weeks before my examinations, I was worried that I could not review all the important contents and the more time I spent studying for final examinations, the more strongly I felt I was going to fail my examinations, as I found that I suddenly could not understand the contents that I thought I had very well learned previously. (Focus group 1#, Student 3#, male).

Except for the challenges students encountered in their study, participants indicated that peer pressure related to their academic performance was another source of stress and anxiety. Involution or neijuan is widespread among Chinese university students, which refers to students’ irrationality and over-competition over higher scores or more resources (Yi et al., 2022). Participants’ anxiety increased due to the pressure that they needed to follow their peers’ pathways or would be perceived as incompetent and incapable. Hence, participants felt concerned and anxious once they thought they lagged behind their peers.

It seems that we only have two options after we graduate: either doing a postgraduate program in China or overseas. If you do not choose between these options, people will regard you as abnormal and unsuccessful. Therefore, I feel quite restrained by competing with my peers to do something I do not like. I have been very anxious as I cannot choose to do what I like, and I dare not to give up pursuing a postgraduate program either. (Focus group 6#, Student 7#, male).

When my roommate studies in our dorm while the rest of us do not, we would be quite anxious, and we would wonder that how come our instructor did not assign any homework to us, which might affect our GPA if we do not do enough exercise. (Focus group 4#, Student 10#, female).

Participants mentioned that the general social atmosphere that supported the involution phenomenon imposed pressure on university students.

I consider that the social atmosphere actually intensifies our generation’s involution. The predominant discourse we are having now is that you have to become an elite, and you need to be the supporting pillar of this country. It seems to me that being normal means being unsuccessful under the current discursive framework. Therefore, I feel lots of pressure and quite depressed. (Focus group 6#, Student 6#, female).

#### 3.2.2 Social influence

The primary reason for students experiencing stress stems from social influence, which incorporates explicitly two distinct aspects, namely, pursuing social acceptance and peer pressure. University students attach significant importance to gaining recognition from their peers and society (Neville et al., 2021). Nevertheless, the pursuit of social acceptance can become a source of stress as students strive to conform to societal expectations and adhere to specific standards or roles, which can be stressful as they are concerned about not being accepted or recognized.

A common reward crisis among my peers and me is our inability to generate a sense of self-worth. In this regard, I rely heavily on others to love, recognize, and praise me. Trying to make myself accepted by others, even in a situation that I feel uncomfortable and stressful. (Focus group 6#, Student 7#, male).

Participants reflected that most students tended to rely on external acknowledgment from others as a critical means to establish their self-worth while being unable to develop a sense of self-confidence and satisfaction from within. However, as the pandemic hit each university harshly, school closures made it impossible for students to socialize in person, which cut off an important channel for students to receive timely social support that helped to build up their sense of being accepted essentially.

I am a person that cannot live without positive feedback from my friends so that I can maintain my positive attitude toward life and study. Therein, when the university closed, and I could not hang out with my friends frequently, I felt very terrible, and it seemed that my source of positive energy had been cut off (Focus group 6#, Student 7#, male).

Concurrently, peer pressure was another source of students’ stress. Comparisons and rivalries often emerge among peers, leading some students to feel inadequate compared to others, thus inducing their feelings of inferiority and stress. A noteworthy interaction existed between social identity and peer pressure, wherein students, upon experiencing peer pressure, might intensify their efforts to align with social expectations in the quest for a sense of identity, thereby further exacerbating their stress levels.

I think the primary source of pressure comes from peer pressure within my friends. I have two amazing friends in my field of study, and we are very close to each other. I feel enormous pressure even though I know comparing myself to others is pointless. We are friends, but at the same time, we are also competitors in a way (Focus group 4#, Student 7#, female).

#### 3.2.3 Pandemic-related factors affected students’ mental health

Changes in students’ lives caused by the COVID-19 pandemic became critical risk factors that endangered students’ mental health and inflicted them with heightened levels of anxiety and stress. The changes in students’ study mode and living arrangements significantly affected their mental health.

Except for the change in students’ study mode, changes in students’ accommodations also affected their mental health. Due to the containing of the spread of the virus, universities would close their campuses, and students needed to stay at home and have online courses. When staying at home, students had a longer time to be with their parents, which caused lots of conflicts and disagreements between them and their parents.

One participant stated that the reason she felt depressed when the university closed was that she had to stay at home and be with her parents for a long period. The intergenerational communication within her family had not been smooth during the process of growing up. Staying with her parents all day long made her feel that she was under close surveillance of her parents, which led to her uneasiness.

I am not used to working with others in the same room, making me uncomfortable. Therefore, when I had to work with my mom in the same room during the lockdown period, I could feel that she closely monitored every move I made, which bothered me severely. We did not have smooth or close intergenerational communication throughout the years that I grew up within my family, so my parents always seemed a bit far away from me, and they felt the same with me, too. When we were forced to stay together and communicate with each other, we all felt awkward and depressed and kept hoping that the pandemic could end immediately. (Focus group 2#, Student 5#, female).

Similar to Participant 5, who had uneasiness with her parents, another participant from the same focus group shared his conflicts with his parents. However, this participant tried to cope with the family stress by shifting their attention to study. Although he had a bumpy relationship with his parents during the lockdown when they were confined in their homes, he made significant progress in his academic performance.

In my case, my conflicts with my parents turned into my motive to study harder. When I was confined at home together with my parents, every little thing could trigger our arguments and conflicts, including the time I got up from bed, I ordered too many takeaways, my intimate relationship, and so forth. Since I could not control the pandemic, I had no idea when the conflicts between my parents and me would finish. What I could control was my own attention, so in order to become too depressed with the conflicts, I shifted my focus to my studies. I had never worked that hard before, as I wanted to numb myself with study and get myself away from the negative feelings inflicted by the conflicts. (Focus group 2#, Student 1#, male).

Besides living with parents due to the lockdown, another situation was that university students were confined to stay on campus and could not leave only if it were a medical or family emergency. On one hand, students were stressed and anxious about this lock-down situation of having to change their plans of traveling or going back home; on the other hand, students had a relatively simple life routine by only moving between their dorms and classrooms under this circumstance, which caused students’ intense relationships, especially with their dormmates, which deteriorated their mental health.

My university had been under lockdown for the last semester. For me, I was not a person who liked to frequently go out and have fun before the pandemic. Therefore, it was supposed not to be a big problem for me if I could not leave campus casually. However, what surprised me most was that I felt very depressed and anxious every time I received a notice from the university informing me that the lockdown measure would continue being implemented until further notice. What bothered me most was that we never had a clear idea of when this lockdown would be over. (Focus group 5#, Student 7#, female).

Though relational problems with roommates were stressors for many students, the campus lockdown caused by the pandemic made it more challenging for students to deal with their relationships with their roommates. The friction caused by different living habits tended to be intensified under lockdown, which imposed stress on students.

COVID-19 has made the relationship among my dormmates really intense. Though we had small bickering before, we always managed to patch up with each other. However, things got worse after the outbreak of the pandemic, maybe because everyone was all stressed out and frustrated, our bickering turned into verbal abuse and even physical attacks. Everyone gets angry easily and tends to turn against each other, even if it is only a very minor issue that might have been ignored previously. One of my dormmates told me that his mental health was severely affected and that he needed to take more medications, which influenced his life and study. (Focus group 1#, Student 3#, male).

What stressed me most and made me anxious was my relationship with my dormmate, who also happened to be my best friend from high school. We were all very depressed by the campus lockdown, but we dealt with our depression in different ways, with me trying to hold back emotions while she lashed out all her anger by frequently throwing or punching things in our dorm, which seriously bothered me. (Focus group 3#, Student 1#, female).

Many students’ schedules, study, and career plans after graduation had greatly changed due to the pandemic, as they needed to cancel their booked plans due to unexpected lockdowns or restrictions and reconsider their job stability. Traveling restrictions made students reconsider their prospective overseas study plan as they might not be able to easily or freely come back. Hence, some participants mentioned that they struggled with their future study plans. On the one hand, these participants wanted to pursue their further postgraduate study abroad; on the other hand, they were concerned about the overseas pandemic situations and, therefore, worried about their personal health, and traveling back to China faced many restrictions.

Because of the pandemic, I might not be able to come back within the next 2 years until I succeed in graduating from my postgraduate study in the U.S. Hence, as a person who is very independent, I feel anxious and nervous about my upcoming trip to America. (Focus group 2#, Student 2#, female).

Unexpected changes in booked plans caused by the pandemic situation brought severe stress and anxiety to some participants as they felt a great disturbance.

I was stressed out by the repetitive flight booking and canceling procedures, and what stressed me more was the possibility of being locked down in the local area and not be coming back to the university on time, which would both be a financial and academic burden for me. I could not fall asleep for days during that period and sensed that I was on the verge of breaking down. (Focus group 3#, Student 10#, male).

Every time I am about to do something, I will start to hesitate and finally give up. This sense of hesitation has made me waste much time and effort, which intensifies my anxiety and stress, as I feel that I have gradually lost control of my own life. (Focus group 2#, Student 8#, male).

## 4 Discussion

This is one of the leading qualitative research that sought to understand Chinese university students’ mental health in the post-pandemic era. Results drawn from six focus groups with 61 participants yielded two categories of knowledge: mental health problems encountered by Chinese university students and the causes of these problems (Table 2).

**Table 2:** Themes and key findings.

Theme	Sub-theme	Key findings
Mental Health Problems	Sleep difficulties	Chinese university students primarily grappled with sleep difficulties, anxiety, and stress as predominant mental health predicaments in the post-epidemic phase.
	Anxiety	
	Stress	
Causes of Students’ Mental Health Challenges	Academic performance	Academic performance posed a serious challenge to participants’ mental health and was closely linked to the cyclicality associated with academic scheduling. Challenges encountered in their studies and peer pressure were the two main areas that contributed to the anxiety and stress felt by the participants.
	Social influence	Students feel anxious about interacting with their peers because they develop social anxiety during periods of social distancing, and social distancing maintains social anxiety in college students. Students are fearful of COVID-19 which is positively correlated with their social anxiety. Students’ excessive pursuit of social approval can also affect their mental health status.
	Pandemic-related factors affected students’ mental health	In the Post-pandemic Era, students will go through a period of readjustment as they endeavor to re-establish their former lifestyles, exercise routines, relationships, and so on.

Three major problems that posed severe challenges to students’ mental health were sleep difficulties, anxiety, and stress. Participants’ sleep difficulties were not particularly induced by the pandemic, rather, this problem was more of a periodic one that was associated with students’ academic arrangement. Participants frequently encountered sleep difficulties during the examination period or their school break. This might be closely associated with their academic pressure, which brought up students’ endless competition with peers and thus gradually raised their own threshold of doing everything until they felt stressed out. This phenomenon reflects the trend of academic involution among Chinese university students, which refers to students’ internal friction and dilemma of undue cost without the corresponding return, and this could elicit typical problems such as fierce competition, educational anxiety, and excessive education (Chen and Bao, 2022). In concordance with our results, a study conducted among college students in the United States unveiled alarming levels of depression, anxiety, and suicidal ideation, highlighting the profound toll of a pandemic on students’ mental health (Wang et al., 2020). Our results echo previous studies indicating that sleep problems were one of the most prevalent mental health challenges among Chinese university students during the pandemic (Ge et al., 2020). However, different from previous research concluding that social distancing negatively affected university students’ sleep quality, we found that academic stress was highly associated with students’ sleep problems rather than social distancing, probably because the studies were carried out at different development stages of the pandemic. As we step into the post-pandemic era, social distancing and closure of schools do not frequently happen, and thus, social distancing would no longer become a primary cause of students’ sleep problems.

Anxiety was the most prevalent mental health problem among participants in this study, which is consistent with prior research (Bandelow and Michaelis, 2022). Due to the strict prevention measures, students were anxious about possible school closure or cancellation of in-person teaching, as they were not accustomed to online teaching, which would lead to low study efficiency and an unbalanced lifestyle. This anxiety issue was closely tied to their academic performance ultimately. In line with our findings, previous research also proposed that academic performance can trigger university students’ anxiety (Guan et al., 2021). A study conducted in Bangladesh during the pandemic’s initial stages underscored students’ contentment with their academic pursuits during the pandemic as a pivotal determinant of their psychological well-being (Islam et al., 2020). This finding aligns with our study’s emphasis on the negative effects of academic stress on students’ mental health, underscoring the significance of fostering a supportive academic enviornment to mitigate adverse psychological repercussions especially during global public health crisis. Moreover, students were anxious about socializing with peers once offline education resumed, as they developed social anxiety during the social distancing period. This supports the previous research concluding that social distancing maintains social anxiety among university students (Arad et al., 2021). Despite the academic performance mentioned above that could cause students’ anxiety, another possible explanation for students’ anxiety is their COVID-19 fear, which is positively related to their social anxiety (Nath Samantaray et al., 2022). Social distancing might reinforce students’ avoidant behaviours toward unwanted social situations and promote students’ negative perspectives on believing that feared situations are strongly scary (Nath Samantaray et al., 2022). In addition, students’ COVID-19 fear was a response to environmental factors like counter-infection measures and pandemic severity, which reshaped students’ individual perceptions of psychological distancing (Zheng et al., 2020).

The findings of this study resonate with those of an international study, which asserted a surge in students’ anxiety, particularly concerning academic pursuits and the fear of COVID-19 (La Rosa and Commodari, 2023). Conversely, our examination unearthed a more conspicuous impact of academic stress on the quality of sleep among Chinese students. This divergence underscores the imperative of probing into cultural and contextual factors when investigating the intricate terrain of mental health dynamics amidst pandemics. Our results echo those of this study as we both found the negative effects of pandemic-induced restrictions on students’ academic and social engagement. The role of social support systems and coping mechanisms in ameliorating students’ stress in response to unprecedented challenges should be further strengthened (Commodari et al., 2021). Moreover, our study sheds light on stressors specific to China, such as heightened competition in the realms of job prospects and higher education, accentuating the necessity for interventions tailored to address varied stressors within a distinctive cultural and socio-economic milieu.

Our findings are consistent with prior research suggesting that stress was prevalent among Chinese university students after the COVID-19 pandemic (Wang et al., 2023). We found that students’ stress could negatively affect their physical and mental health, resulting in poor sleep quality, poor physical health, and deteriorated mental illness with inappropriate coping strategies, which is supported by existing studies (Ribeiro et al., 2018). The COVID-19-related stressors accounted for students’ stress issues. First, pursuing acceptance from their surroundings was a stressor for university students. However, school closures due to the control of the pandemic made it difficult for students to access their friends for timely and in-person social support, which was essential to their feeling of being accepted and thus caused their increased stress level.

Secondly, university students typically relied on social activities, sports, club participation, and similar avenues to alleviate study and life-related stress. However, school closures restricted their access to such stress-relieving opportunities, culminating in the continuous accumulation of stress. Particularly, students accustomed to regular exercise faced limited options, leading to physical and mental health concerns (Wilson et al., 2021). The post-closure phase will witness a readjustment period for students as they endeavor to reestablish their previous lifestyle and exercise habits. Moreover, the closure measures and the transition to online classes have prolonged the cohabitation with roommates or family members. This prolonged proximity has resulted in frequent frictions and conflicts, consequently straining relationships between students and their cohabitants. The persisting tension arising from these conflicts can further impede interpersonal relationships and contribute to increased stress levels (Hall and Zygmunt, 2021). Furthermore, the pandemic’s impact on the job market and higher education prospects has created a sense of uncertainty and fiercer competition among students, which results in intensified peer pressure and more mental health challenges (Tomlinson et al., 2022).

Social media has become a key information source during the pandemic, and the use of social media has grown dramatically on specific occasions, such as natural disasters and crises (Gottlieb and Dyer, 2020), as information must be supplied on time. The need for information about the development of the epidemic and online learning has increased college students use of social media during the epidemic; however, social media use and overexposure to public health information may cause burnout and depressed feelings, which would intensify university students’ negative emotions and exacerbate their mental health problems due to the epidemic (Zhao and Zhou, 2020; Haddad et al., 2021).

Furthermore, these studies collectively advocate for proactive measures aimed at fortifying students’ resilience and well-being in the post-pandemic landscape. The clarion call for robust university psychological services resonates consistently across these inquiries, underscoring the imperative of concerted efforts between educational institutions, policymakers, and public health entities in prioritizing the mental health needs of students.

## 5 Conclusion

This is one of the few studies that employ a qualitative methodology to delve into the mental health problems encountered by Chinese university students in the post-pandemic era, alongside exploring their underlying causative factors. The present study discerned that at the post-epidemic stage, Chinese university students primarily grappled with sleep difficulties, anxiety, and stress as predominant mental health predicaments. Three causes including academic performance, social influence, and pandemic related factors led to the aforementioned mental health challenges encountered by university students.

The results of the study can inform the development of mental health promotion and education activities for university students to bolster their cognitive grasp and cope with mental health problems. Meanwhile, results from this research could illuminate the services stakeholders provide to university students in the future. At the university level, educational institutions should learn from this pandemic and provide education and mental health services to increase students’ resilience and psychological flexibility, implementing interventions that are precisely calibrated and commensurate with the problems encountered by students. Enhancing students’ mental health literacy is essential to help students maintain their well-being when they are in difficult situations like a pandemic and is also beneficial to build up collective empowerment for peers to show support to each other. Moreover, public health policymakers need to take the psychological status of university students as a direct response to epidemic prevention and control policies. Thus, they should develop and implement rapid-response public health emergency measures by taking students’ well-being into consideration paying attention to the needs and distress of different groups of people.

Despite its important contributions, this research still has several limitations. First, participants of this study were from one university, which potentially introduces a bias that hinders the extrapolation of findings to encapsulate the general spectrum of mental health problems and their causative antecedents experienced by university students across diverse institutional settings and geographical contexts. This study took place in a second-tier city in China, where COVID-19 pandemic-related public health situations and policies embodied nuances from those in other areas, thus our results might not be generalized to represent university students’ experiences during the post-pandemic era of different tier cities. Further, no strong relationship was perceived between participants’ socioeconomic backgrounds and their mental health outcomes due to the relative homogeneity of the study group. Secondly, the inherent dynamics intrinsic to the methodology of data collection through focus groups could introduce an interactive influence, thereby potentially influencing and shaping the participants’ responses and perspectives. Future studies should be conducted with students from different universities and regions with various socioeconomic backgrounds to improve the representativeness of the findings and also detect group differences. Quantitative studies should be carried out to further testify the findings of this research.

## Data availability statement

The raw data supporting the conclusions of this article will be made available by the authors, without undue reservation.

## Ethics statement

The studies involving humans were approved by BNU-HKBU United International College Committee on Human Research Protection. The studies were conducted in accordance with the local legislation and institutional requirements. The participants provided their written informed consent to participate in this study.

## Author contributions

XN: Conceptualization, Funding acquisition, Methodology, Writing – original draft, Writing – review & editing. XL: Data curation, Methodology, Writing – original draft, Writing – review & editing. SG: Data curation, Writing – original draft.
