# Supplementary material for: Researching into Chinese university students’ mental health in the post-pandemic era – problems and causes
Source: Front Psychol. 2024 Jun 25;15:1393603. doi: 10.3389/fpsyg.2024.1393603 (PMC11231423; doi:10.3389/fpsyg.2024.1393603)
Supplement: Supplementary file 1 [file Data_Sheet_1.docx]

**Appendix 1**

Discussion Group Interview Guide - Students

EMBRACE Implementation Study

Contextual Analysis

***Note:*** *Text written in bold, italicized capital letters is an additional prompt to be used only to guide the focus group facilitator.*

***[It will take approximately 20 minutes to complete the introductory portion of the focus group.]***

1. **Introduction and Description:**

Hello, my name is XXX, and I am a member of the research team for the EMBRACE project. Thank you for joining this focus group. As indicated in the consent form, we aim to understand the mental health needs of college students at UIC. We are interested in their overall mental health and well-being, the factors influencing their access to social supports and services, and the accessibility and disparities in mental health resources. Your perspectives and ideas, based on your personal or professional experiences, are invaluable. The information gathered here will help adapt and implement Acceptance and Commitment to Empowerment therapy. The questions I ask will guide us in mapping the mental health needs, referral pathways, and available services for college students at UIC.

***[Roll call based on name tags - tabletop number cards with insertable pseudonyms were placed at each seat; and participants were reminded that for the purposes of the focus group study, the appropriate number (or pseudonym) would be used to refer to them].***

First, let's review the informed consent and survey forms in front of you.

***[Moderator: Read the informed consent form carefully and answer all relevant questions. Collect signed consent forms and ensure participants have a copy of this information.]***

***Confidentiality: (Read aloud)***

*Before we begin discussing mental health issues among college students, I'd like to review* ***confidentiality*** *and some basic principles for our discussion today:*

- Everyone's perspective is welcome and important.
- We must keep everything shared in this room confidential and not discuss it outside of this group.
- Confidentiality is effective only if everyone adheres to this agreement.
- You decide what you want to share.
- Anything heard in this room should stay here.
- To ensure everyone's voice is heard, we ask that only one person speaks at a time.
- We will do our best to ensure everyone has a chance to speak.
- This discussion will last about ninety minutes, so please stay on topic.

**Use of Audio Recorders:**

- As stated in the consent form, this group discussion will be audio-recorded for accuracy and to capture as much as possible of everyone's contributions.
- All recordings and transcripts will be securely stored in a password-protected or encrypted drive, locked in the principal investigator's office.
- Identifiable personal information will be removed from the transcripts.
- Only the research team and designated researchers will have access to the transcribed text.
- To facilitate transcription and data analysis, please state your number before answering questions.
- One of us will be taking notes on key points to help explore each person's perceptions further and assist in data analysis.

1. **Interviews:**

- After the moderator asks the first question, the discussion will begin.
- The discussion will respond to participants' general responses to each question.
- **Interview Questions:**

1. **The following questions will help us better understand how students understand mental health, mental illness, and related myths and stigmas.**

I: What comes to mind when you hear the term "mental health"?

II: What about the term "mental illness"?

Enquiry:

- Where did you get these ideas and information?
- Which of these ideas do you agree or disagree with?

Note: How participants agree and/or disagree with each other.

Note: How participants distinguish between mental health and mental illness.

1. **The following questions will help us to better understand common issues related to mental health and well-being that college students face:**

I: What are some of the common mental health issues/needs that college students face? What are some of the mental health challenges that you all face, especially during the epidemic?

II: What are some of the factors that influence these needs?

Probe:

- As participants name problems, ask them to provide specific examples (e.g., anxiety, stress, loneliness, insomnia, addictions-drinking, smoking, sex, video games, internet, etc.).
- Some areas to explore are life needs/support (school, finances, family, work); relationship needs/support (self-perception, family, dating/love, gender, peers, teachers/mentors, etc.), socio-cultural needs/support (gender roles, sexuality, societal rules and expectations; stigmas and taboos), environmental needs/support (proximity to home and living conditions), etc.

1. **The following questions will help us better understand how college students understand and respond to their mental health issues/needs.**

I: If one of your peers or classmates was experiencing mental health challenges, what would she/he do? (Begin this general question by starting with his peers and then move to inquiring about the participants themselves)

Probe:

- Who would be the first person to recognise this student's mental health problem?
- What would this student do? What prompted them to respond in this way? Or why would they respond in this way?
- Who would this student turn to for help and why?
- What types of mental health help or services are available for college students?
- Ask about formal and informal (peers, friends, etc.) support separately.
- How easy or difficult is it for students to seek help, get support, or access these services for their mental health condition?
- What might motivate or discourage students from seeking help or services for their mental health?

**II: What about you? What would you do if you were experiencing a mental health challenge? What are some other resources you would be able to use? Or what are some other barriers you would encounter?**

**(4) The following questions will help us better understand college students' perceptions of improving their access to mental health support and services.**

**I: In your opinion, what types of programs, supports, or services would promote college students' mental health and address their mental health needs?**

**II: What would promote college students' access to these programs or services?**

Probe:

- Mental health promotion and illness prevention activities (e.g., mental health literacy, awareness, stigma reduction, stress reduction, social support, etc.)
- Programs and services to address mental health challenges (on-campus, off-campus, type of support - structured peer support; counseling; psychiatric/medical; online; social media, etc.)

**(5) The main purpose of the EMBRACE program is to engage university students in training that enhances their mental health literacy and promotes the mental health of their peers and other university students. This training consists of three components: 1) an online self-study module on mental health and illness; 2) a series of online workshops to improve mental resilience and wellness; and 3) a mentorship program to support a cadre of participating students in implementing mental health promotion activities for campus and peer support. With that clear:**

I: What do you think are the key components/contents that should be included in an online training module?

[Probe for information on determinants of mental health; information on mental illness - symptoms, treatment, support; available services and resources; self-care strategies, etc.].

II: How important is the emphasis on gender for this training? Would it have been better, at least for some of the workshops, to group them by gender (girls' seminars and boys' seminars)?

III: What are the most effective learning strategies for engaging college students?

[Probe: sharing and discussion, experiential learning, videos/films, guest speakers, situational cases, role-playing, online learning, social media, etc.]

IV: What are some important topics to integrate into face-to-face psychoeducation?

[Inquiry: family, self-awareness, peer relationships, dating/love, addictions, stress and coping, life demands, etc.]

V: What are the most effective strategies to use to recruit students for this program and training?

Probe:

- Where is it? How is it conducted? Types of recruiting messages? Social media strategy?
- What are potential facilitators and barriers?

**(6) Any additional comments on services and supports available to college students with mental health needs? Or training topics and strategies on promoting mental health literacy among college students.**

**Closing Words:**

·Remind participants to "keep their words in the room"

·Thank you to the participants
